# Antistress Effects of *San-Huang-Xie-Xin* Decoction on Restraint-Stressed Mice Revealed by ^1^H NMR-Based Metabolomics and Biochemistry Analysis

**DOI:** 10.1155/2019/5897675

**Published:** 2019-04-16

**Authors:** Wei Peng, Huan Du, Guangli Liu, Qing Zhang, Tingting Kuang, Zhang Wang, Gang Fan

**Affiliations:** ^1^School of Pharmacy, Chengdu University of Traditional Chinese Medicine, Chengdu 611137, China; ^2^School of Ethnic Medicine, Chengdu University of Traditional Chinese Medicine, Chengdu 611137, China

## Abstract

*San-Huang-Xie-Xin* decoction (SHXXD), composed of Rhei Radix et Rhizoma, Coptidis Rhizoma, and Scutellariae Radix, is a representative antipyretic and detoxifying prescription in traditional Chinese medicine. In this study, we investigated the antistress effects and underlying mechanisms of *San-Huang-Xie-Xin* decoction (SHXXD) on restraint-stressed mice by ^1^H NMR-based metabolomics combined with biochemistry assay. A total of 48 male mice (5 weeks old, 18-22 g) were divided randomly into 6 groups (*n* = 8), including the normal group, restraint-stressed group, vitamin C group (positive drug, 17 mg/kg), and 3-dosage groups of SHXXD (200, 400, and 800 mg/kg). The stress model was induced by restraining mice in a polypropylene centrifuge tube for 6 h every day. The rotarod test was performed, and several biochemical indicators were measured. Moreover, other 24 animals were divided into 3 groups (*n* = 8) including the normal group, restraint-stressed group, and SHXXD group (800 mg/kg) for ^1^H NMR-based metabolomics analysis. Our results showed that SHXXD significantly increased the rotarod time, thymus index, spleen index, and the levels of glutathione peroxidase (GSH-Px), superoxide dismutase (SOD), and interleukin- (IL-) 2, but decreased the levels of malondialdehyde (MDA), IL-1*β*, tumor necrosis factor- (TNF-) *α*, corticosterone (CORT), and adrenocorticotropic hormone (ACTH) in restraint-stressed mice. Moreover, the contents of eight endogenous metabolites that were changed by restraint stress were significantly reversed by SHXXD. The results of both metabolomics and biochemical analysis indicated that SHXXD (800 mg/kg, p.o.) could improve the biochemical changes and metabolic disorders in restraint-stressed mice by antioxidation and anti-inflammation, enhancing the body's immune function and restoring several disturbed metabolic pathways (i.e., lipid metabolism, glycolysis and gluconeogenesis, inflammatory injury, and energy metabolism). Taken together, these results indicated that SHXXD has a potential antistress effect in restraint-stressed mice and could be considered as a candidate drug for stress-related disorders.

## 1. Introduction

Previous reports have indicated that stress could negatively affect physical and mental performances of a human being and result in various stress-related disorders, such as anxiety and insomnia [1, 2]. Importantly, it is reported that depression and anxiety, two substantive public health problems, which often lead to heavy social burdens, have the high incidences under stress exposure [3, 4]. However, the current available pharmacotherapies to treat these stress-related disorders are limited due to low efficacy and oppressive side effects [5]. In addition, increasing scientific evidences have suggested that traditional Chinese medicines (TCMs), which have been utilized to treat diseases for thousands of years in China, possess promising curative effects for treating some difficult miscellaneous diseases, including some neuropsychiatric disorders such as depression, anxiety, and insomnia [6, 7].

*San-Huang-Xie-Xin* decoction (SHXXD), composed of Coptidis Rhizoma, Rhei Radix et Rhizoma, and Scutellariae Radix, is a representative antipyretic and detoxifying TCM prescription and first recorded in *Synopsis of Golden Chamber* (a known TCM monograph written by Zhang Zhongjing) [8]. Current phytochemical researches have revealed that the main chemical constituents of SHXXD are alkaloids, anthraquinones, and flavonoids, including berberine, coptisine, palmatine, baicalin, wogonoside, jatrorrhizine, baicalein, aloe-emodin, rhein, and emodin [9, 10]. In addition, previous investigations reported that SHXXD has various pharmacological effects, such as anti-inflammatory, antioxidant, antimicrobial, antiatherosclerosis, and antitumor activities [11–13]. Besides, there has been a clinical report of mental regulative effects of SHXXD [14]. However, to the best of our knowledge, there is no systemic research regarding the effects of SHXXD against stress-related disorders.

Previous evidences revealed that stress possesses detrimental influences on the functions of several cells due to free radical damage, leading to various oxidative stress-related neuropsychiatric diseases [1]. Moreover, previous studies reported that SHXXD could inhibit oxidative stress and inflammation injury of global cerebral ischemia-reperfusion rats [14, 15]. Consequently, we come to the hypothesis that SHXXD might have beneficial effects against restraint stress-induced disorders, and the possible mechanisms might be correlated to its antioxidant and anti-inflammatory properties. This study was designed to evaluate the antistress effects and underlying mechanisms of SHXXD on restraint-stressed mice by using the ^1^H NMR-based metabolomics and biochemistry assay methods, which will not only facilitate the understanding of the pathological changes of restraint stress but also provide valuable information for revealing the antistress effects of SHXXD.

## 2. Materials and Methods

### 2.1. Animals and Ethics Statement

A total of 72 male ICR (Institute for Cancer Research) mice (5 weeks old, 18-22 g) were purchased from the Chengdu *Dashuo* Biological Technology Co. Ltd. (Chengdu, China). All animals were housed in standard mouse cages (29 cm × 17.8 cm × 16 cm) at 21 ± 1°C under a 12 h light/dark cycle and had free access to standard pellet diet (Purina Chow) and tap water, and each cage contains 8 mice. Among these animals, 48 mice were used to evaluate the antistress effects of SHXXD, and other 24 mice were used for ^1^H NMR-based metabolomics analysis. All animal protocols were strictly followed in accordance with the National Institutes of Health Guide concerning the Care and Use of Laboratory Animals, and all the experimental protocols were carried out with the approval of the Animal Experimentation Ethics Committee of the Chengdu University of Traditional Chinese Medicine (No. 2016-12).

### 2.2. Chemicals and Reagents

ELISA kits of malondialdehyde (MDA), superoxide dismutase (SOD), glutathione peroxidase (GSH-Px), interleukin- (IL-) 1*β*, IL-2, tumor necrosis factor- (TNF-) *α*, corticosterone (CORT), and adrenocorticotropic hormone (ACTH) were purchased from the Beijing Yonghui Biological Technology Co. Ltd. (Beijing, China). Deuterium oxide (D_2_O) was purchased from the Cambridge Isotope Laboratories Inc. (Cambridge, UK). Acetonitrile purchased from Fisher Chemicals (Pittsburgh, PA, USA) was of chromatographic grade. Deionized water was obtained from a hi-tech water purification system (Henan, China). The standard compounds of aloe-emodin, rhein, jatrorrhizine, palmatine, berberine, baicalin, wogonoside, baicalein, and wogonin were purchased from Chengdu Push Biotechnology Co. Ltd. (Chengdu, China). All other chemicals used in this study were of analytical reagent grade.

### 2.3. Materials and the Preparation of SHXXD

All the herbs were purchased from Sichuan Neautus Traditional Chinese Medicine Co. Ltd. (Chengdu, China) and authenticated by their appearance traits and the thin-layer chromatography method documented in the *Chinese Pharmacopoeia*. The voucher specimens (Nos. 16031701, 16031702, and 16031703 for the Rhei Radix et Rhizoma, Coptidis Rhizoma, and Scutellariae Radix, respectively) were deposited in the College of Ethnic Medicine, Chengdu University of Traditional Chinese Medicine, Chengdu, China. The *Synopsis of Golden Chamber*, a traditional TCM monograph, recorded that SHXXD consists of Rhei Radix et Rhizoma 6 g, Coptidis Rhizoma 3 g, and Scutellariae Radix 3 g. Therefore, Rhei Radix et Rhizoma, Coptidis Rhizoma, and Scutellariae Radix at the weight ratio of 2 : 1 : 1, reaching a total weight of 500 g, were extracted twice with pure water (1 : 10, *w*/*v*) by decocting (each extraction period lasted 1 h). Subsequently, the decoction was filtrated and concentrated under 50°C *in vacuum* using an RE-2000B rotary vacuum evaporators (Shanghai Yarong Biochemical Instrument Co., Shanghai, China), and the dried residues were treated as the SHXXD extracts which were stored at -20°C before use.

### 2.4. High-Performance Liquid Chromatography (HPLC) Analysis of SHXXD

A conventional HPLC method was applied to qualitatively and quantitatively analyze the main chemical constituents of SHXXD extract. Chromatographic analysis was performed on an Agilent 1260 Series LC system (Agilent Technologies, Germany), consisted of an online degasser, a quaternary pump, a diode array detector, an autosampler, and a column thermostat. The samples were separated on an InertSustain C_18_ column (4.6 mm × 250 mm, 5 *μ*m) at a column temperature of 30°C. The mobile phase was acetonitrile (A) and 0.2% phosphoric acid (B) with a gradient elution program of 5–15% A at 0–10 min, 15–20% A at 10–15 min, 20–23% A at 15–40 min, 23–24% A at 40–55 min, 24–35% A at 55–65 min, and 35–45% A at 65–75 min. The flow rate was 1.0 mL/min, and the injection volume was 10 *μ*L. The detection wavelength was set at 254 nm. The concentrations of aloe-emodin, rhein, jatrorrhizine, palmatine, berberine, baicalin, wogonoside, baicalein, and wogonin in SHXXD extract were determined by using the external standard method.

### 2.5. Animal Grouping and Experimental Protocols

The experimental doses of the testing samples were selected based on commonly used clinical doses of SHXXD for human. The commonly used clinical dosage of SHXXD is 20 g/person/day (crude herb equivalent), and this dose converted to mice should be about 2.6 g/kg/day (crude herb equivalent). In addition, the yield of water extract of SHXXD is about 16%, and so the dose for mice regarding water extract of SHXXD is about 400 mg/kg. Consequently, the 400 mg/kg was treated as the middle dose. In this study, we selected the 200, 400, and 800 mg/kg/day as the low, middle, and high dose for mouse experiments, respectively.

**Figure 1:**
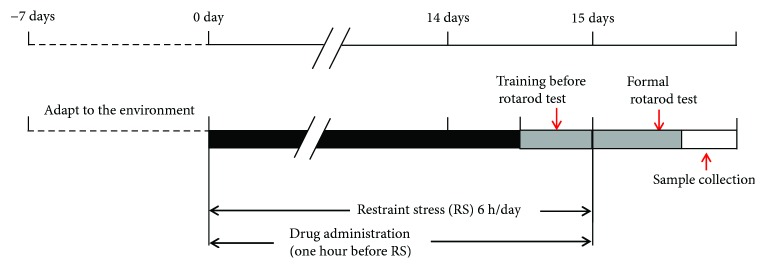
Experimental procedure of the antistress activity evaluation of SHXXD.

After adaption of the environment for 7 days, a total of 48 mice were divided randomly into 6 groups (each group was consisted of 8 mice), including the normal group (normal): normal mice treated with saline; the restraint-stressed group (control): restraint-stressed mice treated with saline; the vitamin C group (VC): restraint-stressed mice pretreated with VC (17 mg/kg/day, p.o.); the SHXXD-L group: restraint-stressed mice pretreated with low-dose of SHXXD (200 mg/kg/day, p.o.); the SHXXD-M group: restraint-stressed mice pretreated with middle dose of SHXXD (400 mg/kg/day, p.o.); and the SHXXD-H group: restraint-stressed mice pretreated with high-dose of SHXXD (800 mg/kg/day, p.o.). All the mice were administered for 14 consecutive days. The restraint-stressed mice were prepared according to the previous reported methods [16]. Briefly, one hour after the administration of VC or SHXXD, all mice except the normal mice were physically restrained in the 50 mL polypropylene centrifuge tube for 6 h once daily lasting for continuous 14 days. Then, the mice received a rotarod test in the 15th day, and blood samples were collected immediately using an orbital blood sampling after the rotarod tests for further ELISA assays, and the mice were subsequently sacrificed by decapitation at the end of the experiment. The brain, thymus, and spleen were dissected from each mouse to determine their visceral indices according to the following formula (1). In addition, brain tissues were also collected for further ELISA assays. The experimental procedure is shown in Figure 1.

(1) $$\begin{array}{c} \%\text{Visceral indices}=\frac{\text{Visceral weight}}{\text{Brain weight}}\times 100. \end{array}$$

### 2.6. Rotarod Test

The rotarod test was carried out according to the previous reported method with minor modifications [17]. After the final 6-hour restraint in the 14th day, the mice were suffered an adaptive training on a rotarod test at a speed of 20 rpm for 2 min by using a ZB-200 rotarod apparatus (Techman Soft Co., Chengdu, China) before the formal rotarod test. On the next day, the standing time on rolling stick of the mice was recorded with a 15 min experimental period and a rotate speed of 30 rpm (each mouse was tested for 3 times, and the average standing time was calculated).

### 2.7. Biochemistry Tests

Serum samples were prepared by leaving whole blood to stand at room temperature for coagulation and then centrifuged for 10 min at 4000 rpm. Brain homogenate 10% (*w*/*v*) was prepared in phosphate-buffered saline (PBS) and centrifuged at 4000 rpm for 10 min under 0°C. All the testing samples were stored at -80°C until ELISA analysis. Furthermore, the contents of CORT, ACTH, IL-1*β*, IL-2, and TNF-*α* in serum and contents of MDA, SOD, and GSH-Px in the brain were determined using commercial ELISA kits according to the manufacturer's protocols and instructions.

### 2.8. Statistical Analysis

Data are represented as the mean ± S.D. One-way analysis of variance (ANOVA) followed by Dunnett's test was done with GraphPad Prism software version 5.0 (GraphPad Software Inc., San Diego, CA, USA) to detect intergroup differences. A probability (*p*) value of less than 0.05 was considered statistically significant.

### 2.9. ^1^H NMR-Based Metabolomics Analysis

#### 2.9.1. Sample Preparation

In view of the good results of the high-dose group of SHXXD in biochemical tests, its serum samples were further used for metabolomics analysis. After adaption of the environment for 7 days, a total of 24 mice were divided randomly into 3 groups (each group was consisted of 8 mice), including the normal group (normal): normal mice treated with saline; the restraint-stressed group (control): restraint-stressed mice treated with saline; and the SHXXD group: restraint-stressed mice pretreated with high-dose of SHXXD (800 mg/kg/day, p.o.). All the mice were administered for 14 consecutive days. Blood samples were collected using an orbital blood sampling after the final 6-hour restraint in the 14th day.

^1^H NMR sample preparation was performed according to the method described in previous investigations with some modifications [18]. Briefly, 200 *μ*L serum samples were added to a 1.5 mL centrifuge tube, and then 100 *μ*L D_2_O and 300 *μ*L NaH_2_PO_4_/K_2_HPO_4_ buffer (45 mM, pH 7.4) were added. Furthermore, the mixture was centrifuged for 10 min at 12,000 rpm, and subsequently, 550 *μ*L supernatant was transferred into a standard 5 mm NMR tube. Finally, the NMR tube was sealed and further applied for ^1^H NMR measurement.

#### 2.9.2. ^1^H NMR Spectroscopy Analysis

All ^1^H NMR spectra were recorded on an Agilent DD2 600 MHz NMR spectrometer (Agilent Technologies, Santa Clara, USA) operating at a proton frequency of 600.13 MHz. ^1^H NMR experiments were performed with the following parameters: relaxation delay, 2.0 s; acquisition time, 1.36 s; number of scans, 64; pulse program, cpmgpr1d (Bruker notation); and spectral width, 12,019.2 Hz.

#### 2.9.3. Data Processing and Statistical Analysis

All the sample spectra were phased and manually baseline corrected using MestReNova software (version 9.0.1, Mestrelabs Research SL). The sample spectra were referenced to a lactate signal at 4.11 ppm. In addition, they were segmented into consecutive nonoverlapping regions of 0.002 ppm chemical shift bins between 0.50 and 9.50 ppm. The region from 4.68 to 5.00 ppm, which contained the residual water signal, was excluded. The spectra were normalized to the total area of metabolites' spectral integrals to compensate for differences in sample concentration. The ^1^H NMR spectra were automatically reduced to ASCII files using MestReNova software. Output data were imported into SIMCA software (version 11.5, Umetrics, Umeå, Sweden) for multivariate statistical analysis. Metabolites that were detected were assigned by referring to relevant literatures [19–24] and querying several databases (Madison, http://mmcd.nmrfam.wisc.edu/; KEGG, http://www.genome.jp/kegg/; and HMDB, http://www.hmdb.ca/).

The Pareto-scaled NMR data were analyzed with nonsupervised principal component analysis (PCA) and supervised partial least squares discriminant analysis (PLS-DA) using SIMCA software (version 11.5, Umetrics, Umeå, Sweden). The parameters of *R*^2^*Y* indicated the validity of the models against overfitting, while the parameters of *Q*^2^ indicated the predictive ability. Moreover, the mean integral areas of bins belonging to the potential biomarkers that were obtained from PLS-DA were calculated and normalized to the total spectral intensity. To determine if changes in bin intensities were statistically significant fold changes, Student's *t*-test was performed using MetaboAnalyst 4.0 (http://www.metaboanalyst.ca/). Altered features were considered significant when pertaining *p* values were less than 0.05.

## 3. Results

### 3.1. The Chemical Constituents of SHXXD Determined by HPLC

**Figure 2:**
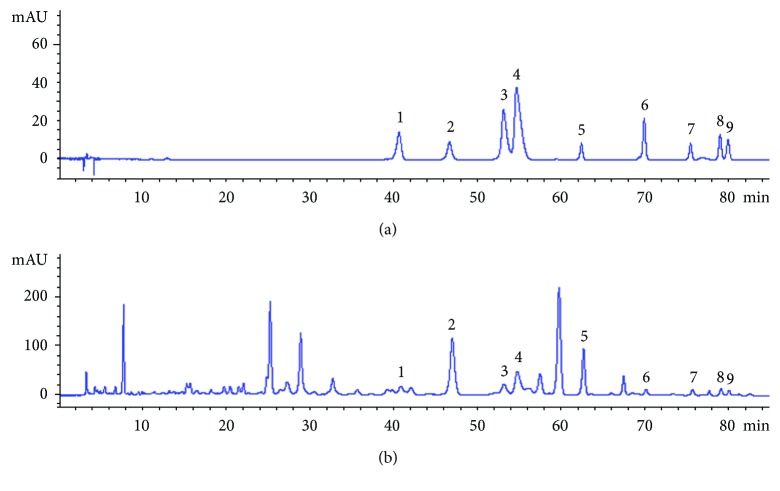
HPLC chromatograms of mixed standard compounds (a) and SHXXD extract (b). Nine chromatographic peaks were identified: (1) jatrorrhizine, (2) baicalin, (3) palmatine, (4) berberine, (5) wogonoside, (6) baicalein, (7) aloe-emodin, (8) rhein, and (9) wogonin.

The HPLC chromatogram of SHXXD extract is shown in Figure 2. A total of 9 constituents including aloe-emodin, rhein, jatrorrhizine, palmatine, berberine, baicalin, wogonoside, baicalein, and wogonin were identified by comparing their retention time and ultraviolet spectra with those of the standard compounds. Among them, aloe-emodin and rhein are mainly from Rhei Radix et Rhizoma; jatrorrhizine, palmatine, and berberine are from Coptidis Rhizoma; and baicalin, wogonoside, baicalein, and wogonin are from Scutellariae Radix. Moreover, the contents of the nine compounds were calculated according to the external standard method. It was found that baicalin showed the highest level in SHXXD extract and its content was 49.63 mg/g, followed by wogonoside (11.99 mg/g). The other 7 compounds (i.e., rhein, aloe-emodin, baicalein, wogonin, jatrorrhizine, palmatine, and berberine) showed low concentrations in SHXXD extract, and their contents are 1.80, 0.89, 1.29, 2.96, 1.94, 3.70, and 5.06 mg/g, respectively.

### 3.2. Effects of SHXXD on the Rotarod Time of Restraint-Stressed Mice

**Figure 3:**
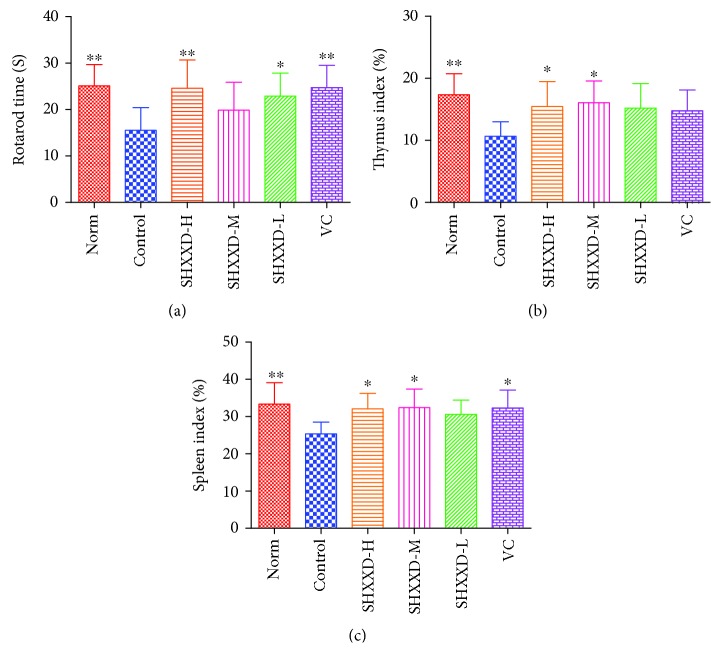
Effects of SHXXD on rotarod time (a), thymus index (b), and spleen index (c). The data are presented as the mean ± S.D. (*n* = 8); ^∗^*p* < 0.05 and ^∗∗^*p* < 0.01 vs. the control group.

As shown in Figure 3(a), after the 14-day restrain stress, the rotarod time of restraint-stressed mice (control group) was significantly decreased compared with the normal mice (*p* < 0.01). However, our results revealed that treatment with vitamin C (*p* < 0.01) and SHXXD at low (*p* < 0.05) and high (*p* < 0.01) doses could significantly increase the rotarod time, compared with the control group.

### 3.3. Effects of SHXXD on Thymus and Spleen Indexes of Restraint-Stressed Mice

Furthermore, the organ indexes of the thymus and spleen were determined in our present study, and the results shown in Figures 3(b) and 3(c) indicated that the thymus and spleen indexes were significantly lower in restraint-stressed mice than those in the normal mice (*p* < 0.01). After treatment with vitamin C, the spleen index was significantly increased (*p* < 0.05) compared with the control group. Similarly, SHXXD treatment at middle and high doses also reversed the decrease in the spleen index (*p* < 0.05), compared with the control group. In addition, administration of SHXXD at middle and high doses could also increase significantly the thymus index (*p* < 0.05), compared with the control group.

### 3.4. Effects of SHXXD on Serum CORT and ACTH of Restraint-Stressed Mice

**Figure 4:**
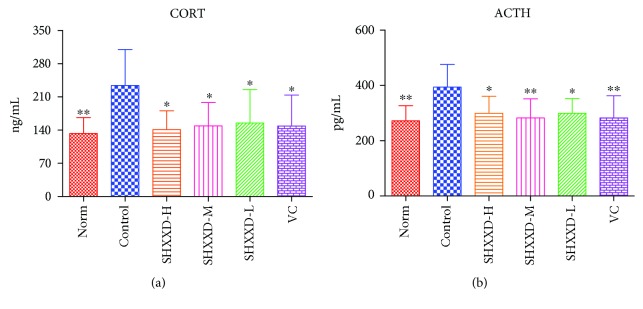
Effects of SHXXD on serum CORT (a) and ACTH (b) levels. The data are presented as the mean ± S.D. (*n* = 8); ^∗^*p* < 0.05 and ^∗∗^*p* < 0.01 vs. the control group.

As can be seen from Figure 4, the levels of CORT (Figure 4(a)) and ACTH (Figure 4(b)) in the serum of the restraint-stressed mice were significantly higher than those in the normal mice (*p* < 0.01). However, our results revealed that vitamin C could significantly decrease the serum contents of CORT (*p* < 0.05) and ACTH (*p* < 0.01), compared with the control group. Similarly, treatment with SHXXD at low, middle, and high doses also significantly reduced the serum levels of CORT (*p* < 0.05) and ACTH (*p* < 0.05), compared with the control group.

### 3.5. Effects of SHXXD on Serum Cytokines of Restraint-Stressed Mice

**Figure 5:**
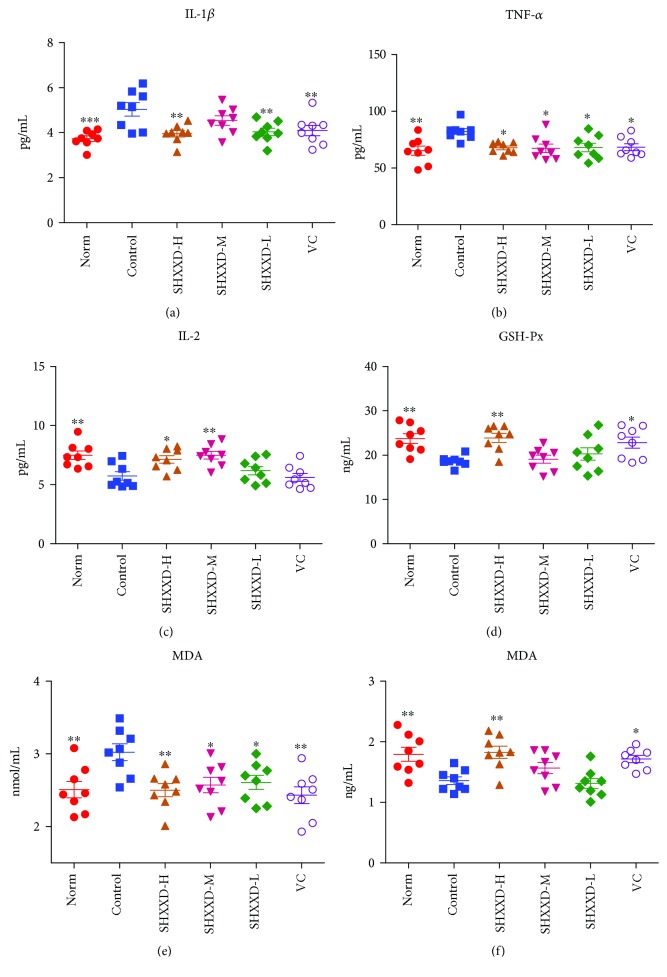
Effects of SHXXD on serum cytokines (IL-1*β* (a), TNF-*α* (b), and IL-2 (c)) and GSH-Px (d), MDA (e), and SOD (f) in the brain of restraint-stressed mice. Each point represents an individual sample. Error bars indicate the mean ± S.D. (*n* = 8). ^∗^*p* < 0.05, ^∗∗^*p* < 0.01, and ^∗∗∗^*p* < 0.001 vs. the control group.

As shown in Figures 5(a)–5(c), the levels of TNF-*α* (*p* < 0.01) and IL-1*β* (*p* < 0.001) were significantly higher, whereas the IL-2 level was significantly lower (*p* < 0.01) in the serum of the restraint-stressed mice than that in the normal mice. However, vitamin C could significantly decrease the levels of TNF-*α* (*p* < 0.05) and IL-1*β* (*p* < 0.01) compared with the control group (Figures 5(a) and 5(b)). Interestingly, similar to the vitamin C, SHXXD at low and high doses could significantly decrease the serum levels of TNF-*α* (*p* < 0.05) and IL-1*β* (*p* < 0.01), and the middle dose of SHXXD could also reduce the TNF-*α* level (*p* < 0.05), compared with the control group (Figures 5(a) and 5(b)). Besides, our results also suggested that SHXXD at middle and high doses could increase significantly the IL-2 level (*p* < 0.05), compared with the control group (Figure 5(c)).

### 3.6. Effects of SHXXD on GSH-Px, MDA, and SOD of Restraint-Stressed Mice

The effects of SHXXD on the levels of GSH-Px, MDA, and SOD in brain tissues of restraint-stressed mice are shown in Figures 5(d)–5(f). After the 14-day restrain stress, the levels of GSH-Px (*p* < 0.01) and SOD (*p* < 0.01) in brain tissues of the restraint-stressed mice were significantly reduced, whereas the MDA (*p* < 0.01) was significantly increased, compared with the normal mice. However, our results revealed that vitamin C treatment could significantly enhance the levels of GSH-Px (*p* < 0.05) (Figure 5(d)) and SOD (*p* < 0.05) (Figure 5(e)) in brain tissues compared with the control group, but reduced the level of MDA (*p* < 0.01) (Figure 5(f)). Similar to the vitamin C, SHXXD at the high dose could significantly increase the levels of GSH-Px (*p* < 0.01) (Figure 5(d)) and SOD (*p* < 0.01) (Figure 5(e)) in brain tissues, compared with the control group. Furthermore, SHXXD at the low, middle, and high doses could significantly reduce the MDA level in brain tissues (*p* < 0.05) (Figure 5(f)), compared with the control group.

### 3.7. Effects of SHXXD on Serum Metabolites of Restraint-Stressed Mice by ^1^H NMR Metabolomics Analysis

**Figure 6:**
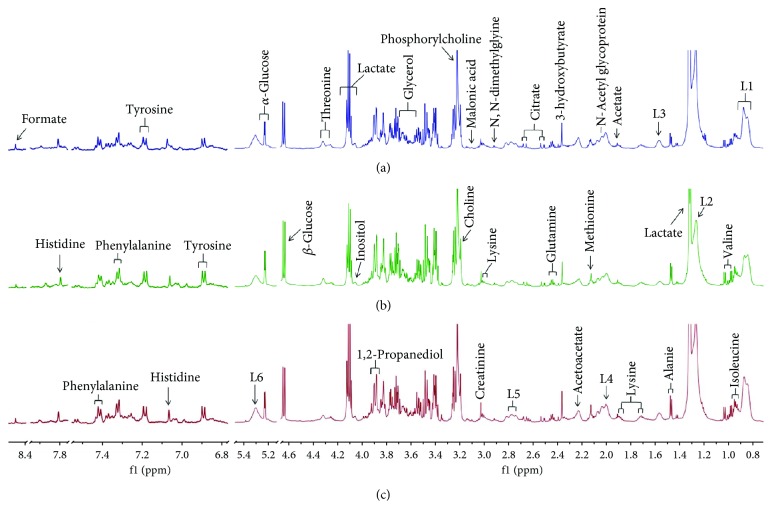
Representative ^1^H NMR CPMG spectra of the serum samples of mice ((a) normal group, (b) SHXXD group, and (c) control group) in the range of 0.8-8.5 ppm. The low-field region (6.8-8.5 ppm) was magnified 12 times from the remaining region for the purpose of clarity. Signal assignments: (L1) LDL and VLDL, CH_3_–(CH_2_)_n_–; (L2) LDL and VLDL, CH_3_–(CH_2_)_n_–; L3 (lipids), –CH_2_–CH_2_–C=O; (L4) lipids, –CH_2_–CH=CH–; (L5) lipids, =CH–CH_2_–CH=; and (L6) lipids, –CH=CH–.

In this study, the ^1^H NMR-based metabolomics method was carried out to study the effects of SHXXD on the serum metabolites of restraint-stressed mice. The representative ^1^H NMR spectra of the serum samples from the normal, restraint-stressed, and SHXXD groups are displayed in Figure 6 with major metabolites labelled. By referencing some literatures [19–24] and comparison with several databases, a total of 29 metabolites were identified including tyrosine, formate, histidine, phenylalanine, *α*-glucose, *β*-glucose, threonine, lactate, inositol, 1,2-propanediol, glycerol, phosphorylcholine, choline, malonic acid, lysine, N,N-dimethylglycine, creatinine, citrate, glutamine, 3-hydroxybutyrate, N-acetyl glycoprotein, methionine, acetoacetate, acetate, alanine, valine, isoleucine, low-density lipoprotein (LDL) and very low-density lipoprotein (VLDL), and lipids (saturated as well as unsaturated).

**Figure 7:**
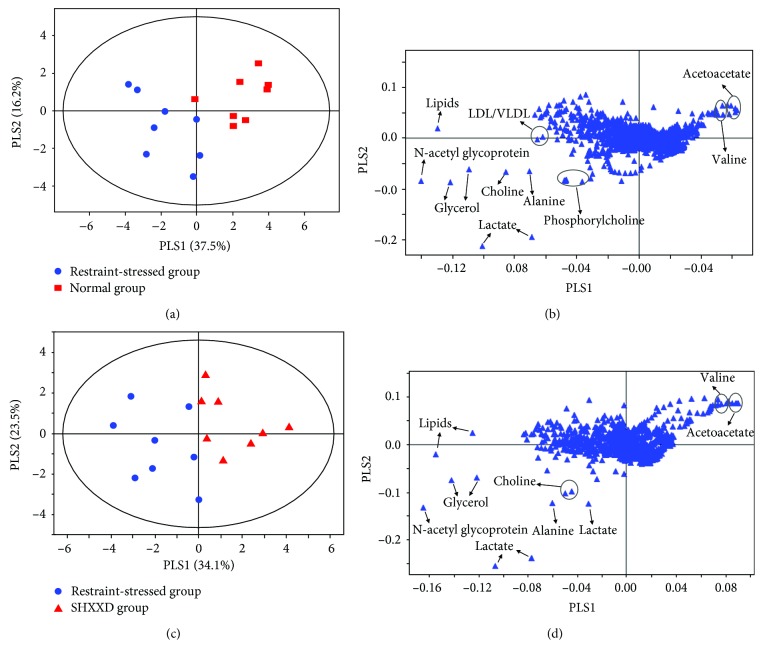
Score plots and corresponding loading plots generated from pairwise PLS-DA analysis of ^1^H NMR data: (a) score plot of the normal and restraint-stressed groups; (b) loading plot of the normal and restraint-stressed (control) groups; (c) score plot of the restraint-stressed and SHXXD groups; (d) loading plot of the restraint-stressed and SHXXD groups.

In order to find patterns in the ^1^H NMR data and differential metabolites, multivariate data analyses were performed. Firstly, PCA, a classical unsupervised approach, was performed to detect patterns in the serum metabolites. The normal, restraint-stressed, and SHXXD groups cannot be clearly separated in the PCA score plot (data not shown). Subsequently, PLS-DA, a supervised pattern recognition method, was further conducted to maximize the separation between groups. The score plot of PLS-DA (Figure 7(a)) showed that this model could reliably differentiate the restraint-stressed group from the normal group (with *R*^2^*X* = 0.850, *R*^2^*Y* = 0.935, and *Q*^2^ = 0.803), suggesting significant metabolic changes induced by restraint stress. Similarly, a clear separation between the control group and the SHXXD group (Figure 7(c)) was obtained by the PLS-DA analysis (with *R*^2^*X* = 0.839, *R*^2^*Y* = 0.898, and *Q*^2^ = 0.757), which indicated that their metabolic profiles were different. Moreover, the corresponding loading plot of PLS-DA revealed the metabolites having significant contributions to the intergroup differences. The loading plot (Figure 7(b)) showed that the separation between the restraint-stressed group and the normal group mainly arose from the signals of lactate, alanine, N-acetyl glycoproteins, glycerol, choline, acetoacetate, LDL/VLDL, lipids, phosphorylcholine, and valine. In addition, eight metabolites (i.e., lactate, alanine, N-acetyl glycoproteins, glycerol, choline, lipids, acetoacetate, and valine) were found to play an important role in separating the SHXXD group from the restraint-stressed group (Figure 7(d)). These results indicated that these metabolites were significantly affected by the restraint-stressed procedure and drug administration and could serve as potential biomarkers.  Table 1 shows these 10 discriminatory metabolites.

**Figure 8:**
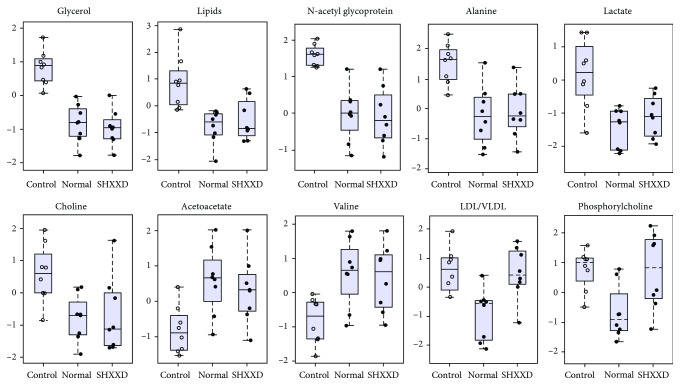
Boxplot of relative levels of the 10 differential metabolites that were obtained from PLS-DA in the serum of the normal, restraint-stressed (control), and SHXXD groups of mice. Data were normalized to the total spectral area, and so the bar plots show the normalized values, and *Y* axes are represented as relative units.

Moreover, in order to validate the PLS-DA results, mean integral areas of these 10 metabolites in normalized spectral bins were calculated and compared in the three groups by the use of Student's *t*-test as reported in Figure 8 and Table 1. The results indicated that lactate, LDL/VLDL, alanine, lipids, N-acetyl glycoproteins, glycerol, choline, and phosphorylcholine levels were significantly increased, whereas acetoacetate and valine were significantly decreased by the restraint stress procedure. These findings were in agreement with several previous reports [22–24]. After drug administration, the levels of these metabolites that were changed by the restraint stress were significantly reversed by SHXXD except for LDL/VLDL and phosphorylcholine. These results suggested that SHXXD has a therapeutic effect in adjusting the changed levels of metabolites in restraint-stressed mice.

## 4. Discussion

Increasing researches have revealed that stress-related mental disorders show the rising incidence due to the intensifying competitions and rapid developing society [25, 26]. Furthermore, herbal medicines are reported be a precious resource for discovering novel drugs for treating various diseases [27, 28]. To the best of our knowledge, this study is the first systemic report on ameliorative effects of SHXXD, a representative antipyretic and detoxifying herbal medicinal prescription in China, against restraint stress-induced symptoms in mice.

Previous investigations have demonstrated that restraint is an ideal and preferred way for preparing stressed animals, and the restraint-stressed mouse model has been extensively used to investigate the pathology of stress-related disorders and to screen the efficacy of candidate antistress drugs [1, 29]. Consequently, we selected the restraint way to prepare the stress mice for our investigation. According to previous studies, vitamin C is a commonly used positive agent for investigations regarding restraint stress in mice [16, 30, 31]. The reasons are due to that vitamin C has a strong antioxidant capacity, and importantly, vitamin C has a good permeability to the brain-blood barrier (BBB) via the sodium ascorbate cotransporters (SVCTs), especially the SVCT2, which is mainly distributed in the brain, eyes, and skeletal muscles [32, 33]. Therefore, we selected the vitamin C as the positive drug to control the present study.

Activation of the hypothalamic-pituitary-adrenal (HPA) axis plays an important role in physiological response of animals to stress and commonly results in immune organs atrophy in stress, such as the thymus and spleen [1, 6]. Furthermore, HPA axis activation could also increase the serum levels of CORT and ACTH. Previous reports have demonstrated that restraint stress could activate the HPA axis, and downregulating the abnormal activation of HPA axis might be beneficial for controlling stress-related disorders [34]. Restraint stress could induce chronic fatigue by overreleasing various hormones such as CORT, ACTH, and catecholamines [35, 36]. The rotarod test is a commonly used way for evaluating the antifatigue effects of candidate drugs [37]. In the present study, we found that SHXXD could decrease the serum levels of CORT and ACTH, but increase the organ indexes of the thymus and spleen. Besides, the results indicated that SHXXD treatment could prolong the rotarod time of the restraint-stressed mice. All these results mentioned above suggested that SHXXD could ameliorate the stress-induced fatigue and reduce the abnormal activation of the HPA axis in stress.

Previous investigations also revealed that besides the immune organs, the serum level of IL-2, released by activated T cells and closely related to the body's immunity, is also reduced by various stress conditions [6]. Interestingly, our results indicated that SHXXD could increase the IL-2 level in the serum of restraint-stressed mice as well as the thymus and spleen indices, suggesting that SHXXD could enhance the body's immunity of restraint-stressed mice. It is reported that increased releases of proinflammatory cytokines such as TNF-*α* and IL-1*β* commonly appeared in individuals with stress-related disorders [38], and the present results revealed that SHXXD could suppress the overexpressed proinflammatory cytokines of TNF-*α* and IL-1*β*. Stress has harmful effects on cell functions via damaging antioxidant defense system, resulting in oxidative and finally leading to various oxidative stress-related diseases [39]. It is reported that GSH-Px and SOD are two major intracellular antioxidants for antioxidative stress [30]. Lipid peroxidation is recognized as a potential molecular mechanism of cell or tissue damages during oxidative stress, and reactive oxygen species in the brain induced by stress could result in lipid peroxidation [40]. MDA is one of the major end products of lipid peroxidation and is commonly used as an indicator for oxidative injury [41]. Our present findings revealed that SHXXD could increase the levels of GSH-Px and SOD, but decrease the MDA in brain tissues of restraint-stressed mice, indicating that SHXXD might have potential antioxidant activity, which is beneficial to maintain the normal antioxidant defense system.

Metabolomics is a useful strategy for investigating the holistic metabolic changes and potential metabolic pathways of TCMs *in vivo* [21, 23]. In this study, the effects of SHXXD on restraint-stressed mice were investigated for the first time using a ^1^H NMR-based metabolomics approach combined with statistical analysis. The results indicated that SHXXD treatment could change the endogenous metabolites in restraint-stressed mice, and the main biomarkers are lactate, alanine, N-acetyl glycoproteins, glycerol, choline, lipids, acetoacetate, and valine. Furthermore, by analyzing the biochemical processes of these biomarkers, we found that the antistress effects of SHXXD might be predominantly related to the glycolysis and gluconeogenesis, lipid metabolism, inflammatory injury, and energy metabolism.

Alanine and lactate play important roles in various biochemical processes. Lactate is the end product of glycolysis. The two metabolites are easily converted from pyruvate by alanine aminotransferase (ALT) and lactate dehydrogenase (LDH), respectively [19]. Some studies have found that restraint stress can cause a significant increase in serum ALT and LDH activities [42, 43]. In addition, chronic restraint stress for 30 days could impair glucose metabolism in rats [44]. Therefore, in the present study, the higher levels of alanine and lactate were observed in the serum of restraint-stressed mice than those in normal mice, which might mean a disorder of glycolysis and gluconeogensis induced by restraint stress. The increased alanine level might be caused by the transformation of acetyl-CoA, while gluconeogenesis from amino acids was suppressed [20]. Moreover, the increase of serum lactate was related to anaerobic cell respiration, which is a kind of energy metabolism process. After SHXXD intervention, the results showed a tendency of bringing the levels of alanine and lactate back to normal, suggesting that SHXXD could adjust the disorder of glycolysis and gluconeogenesis caused by restraint stress. Choline, an important membrane phospholipid constituent, was observed to be increased significantly in the restraint-stressed mice compared with the level in the normal mice, which would be the evidence that lipid metabolism and membrane fluidity were disrupted. Besides, considerably increased levels of glycerol and lipids were observed in the restraint-stressed mice, suggesting a dysfunction of lipid metabolism. After SHXXD intervention, the levels of glycerol, lipids, and choline were adjusted back to normal, which suggested that SHXXD could alleviate the lipid metabolism disorder caused by restraint stress. N-acetyl glycoproteins are acute-phase reaction glycoproteins, which are related to the inflammatory response [22]. They increased when the body was subjected to external or internal challenges, such as infection, inflammation, or stress [21]. In the present study, the increased N-acetyl glycoproteins in the restraint-stressed mice provided evidence for an inflammatory response resulting from the restraint stress procedure. Moreover, after SHXXD treatment, the level of N-acetyl glycoproteins was remarkably attenuated, indicating that SHXXD could alleviate the inflammatory injury. The findings were consistent with the above biochemical test results that SHXXD had significant anti-inflammatory activity. Acetoacetate, a well-known ketone body, is produced by the liver from fatty acids for the body to use as energy [23]. In this study, acetoacetate was observed to be decreased significantly in the restraint-stressed mice compared with the level in the normal group, suggesting that a disorder of energy metabolism may have been present in restraint-stressed mice. In addition, valine plays an important role in the TCA cycle after transformation into succinyl-CoA [24]. In the present study, the level of valine was significantly reduced in restraint-stressed mice measured by ^1^H NMR, indicating an impairment of the TCA cycle. Moreover, the increase in serum lipids indicated a reduction of energy production. After SHXXD treatment, acetoacetate, valine, and lipid levels all show a tendency to return to normal status. These changes suggested that SHXXD could regulate the energy metabolism of the restraint-stressed mice and maintain the energy balance of the body.

## 5. Conclusion

In conclusion, our present study firstly investigated the antistress effects of *San-Huang-Xie-Xin* decoction (SHXXD) on restraint-stressed mice *in vivo*. The results revealed that SHXXD could ameliorate the restraint stress-induced disorders, and the underlying mechanisms may be related to its antioxidation and anti-inflammation, enhancing the body's immune function and restoring several disturbed metabolic pathways (i.e., lipid metabolism, glycolysis and gluconeogenesis, inflammatory injury, and energy metabolism). Further investigations are needed to fully elucidate the antistress mechanism of SHXXD.

## Figures and Tables

**Table 1:** Potential biomarkers in mouse serum and their variations among groups.

No.	Metabolite	Chemical shift (ppm)^a^	Assignment	Control vs. normal^b^	SHXXD vs. control^b^
1	LDL/VLDL	0.87 (br), 1.27 (br)	–CH_3_, –CH_2_	↑^∗^	—
2	Lactate	1.32 (d), 4.11 (q)	*β*–CH_3_, *α*–CH	↑^∗∗^	↓^∗∗^
3	Alanine	1.47 (d)	*β*–CH_3_	↑^∗∗^	↓^∗∗^
4	Lipids	1.57 (br)	–CH_2_–CH_2_–C=O	↑^∗∗∗^	↓^∗∗^
5	N-acetyl glycoproteins	2.04 (s)	–CH_3_	↑^∗∗∗^	↓^∗∗∗^
6	Glycerol	3.56 (m), 3.69 (m)	–CH_2_, –CH_2_	↑^∗∗∗^	↓^∗∗∗^
7	Choline	3.20 (s)	–N–(CH_3_)_3_	↑^∗^	↓^∗^
8	Phosphorylcholine	3.22 (s)	–N–(CH_3_)_3_	↑^∗^	—
9	Acetoacetate	2.23 (s)	–CH_3_	↓^∗∗^	↑^∗^
10	Valine	0.98 (d), 1.03 (d)	*γ*–CH_3_, *γ*–CH_3_	↓^∗^	↑^∗^

^a^s: singlet; d: doublet; q: quartet; m: multiplet; br: broad resonance. ^b^Metabolites with “↑/↓” means increased/decreased; “—” means no significant differences. ^∗^, ^∗∗^, and ^∗∗∗^ indicate *p* < 0.05, *p* < 0.01, and *p* < 0.001, respectively.

## Data Availability

The data used to support the findings in this paper are available from the corresponding author upon request.
